# Commentary: Resistance breakers: a novel approach to tackle biofilm associated infections and antimicrobial resistance

**DOI:** 10.3389/fmicb.2026.1976224

**Published:** 2026-10-08

**Authors:** Wenjie Guo, Ke Xue

**Affiliations:** 1 College of Life Engineering, Shenyang Institute of Technology, Fushun, China; 2 Department of Agriculture and Animal Husbandry, Fuxin Higher Vocational College, Fuxin, China

**Keywords:** antibiotic adjuvant, antimicrobial resistance, resistance breaker, susceptibility restoration, synergy

## Introduction

Nakazwe et al. (2026) bring matrix disruptors, efflux pump inhibitors, enzyme inhibitors, membrane permeabilizers, metabolic modulators, and quorum-sensing inhibitors under the broad term resistance breakers. This inclusive framework is useful for surveying strategies that may enhance antibiotic activity. We do not seek to replace that umbrella definition or exclude any of these categories. Instead, we propose a narrower operational standard for experimental validation and more precise terminology for adjuncts acting through distinct mechanisms. Under this standard, resistance breaking is a pair- and state-specific claim involving the adjunct, partner antibiotic, bacterial resistance phenotype or determinant, and physiological state.

## Resistance breaking depends on the antibiotic pair and bacterial state

Resistance, tolerance, and persistence can all contribute to treatment failure but support different claims. Resistance is a population-level reduction in susceptibility, usually reflected by a higher MIC and maintained during growth, whereas tolerance denotes slower killing without a corresponding MIC increase; persistence describes survival of a subpopulation during antibiotic exposure (Balaban et al., 2019). This distinction separates several examples reviewed by Nakazwe et al. (2026). A beta-lactamase inhibitor can reverse a defined enzymatic resistance mechanism. By contrast, carbon sources that increase aminoglycoside uptake in dormant or persister-like cells primarily modify a physiological state (Allison et al., 2011), while matrix disruptors improve antibiotic access and membrane-active compounds may contribute directly to killing.

The review also provides direct evidence that the partner antibiotic matters. Cinnamaldehyde enhanced colistin activity against Pseudomonas aeruginosa biofilms but did not show checkerboard synergy with carbenicillin, tobramycin, or erythromycin; its interaction with tobramycin was described as additive in biofilm assays (Nakazwe et al., 2026). Similarly, the review notes that the efflux inhibitor CCCP affects proton motive force and bacterial metabolism, and that its interaction with carbapenems may be independent of efflux inhibition. These examples support evaluating the complete adjunct-antibiotic-organism-state combination rather than assigning a fixed resistance-breaker identity to the adjunct alone.

## Evidence should match the claim and physiological state

Checkerboard and time-kill assays answer different questions. Checkerboard testing classifies growth-inhibitory interactions at a defined endpoint; its fractional inhibitory concentration index should therefore be interpreted using pre-specified criteria, because thresholds and the classification of additive or indifferent interactions vary among studies. A reduced antibiotic MIC during combination exposure is an observed susceptibility shift, not proof of enhanced killing. Time-kill experiments assess killing dynamics. Synergy is commonly defined as a reduction of at least 2 log10 CFU/mL relative to the most active single agent, whereas bactericidal activity is commonly defined as a reduction of at least 3 log10 CFU/mL from the starting inoculum. These thresholds and the assessment time should be specified. Disagreement between the methods can reflect their different endpoints and does not invalidate either approach (White et al., 1996). True resistance reversal additionally requires an initially resistant phenotype and evidence connecting the shift to the relevant resistance mechanism.

Clinical breakpoints require equally careful wording. If the antibiotic MIC measured with an adjunct falls below a breakpoint established for antibiotic monotherapy, the result should be reported as: “the combination reduced the MIC to a value below the monotherapy clinical breakpoint.” It should not be interpreted as formal susceptibility classification of the antibiotic-adjunct combination unless combination-specific criteria exist. Monotherapy breakpoints do not capture adjunct exposure, combined toxicity, or dosing requirements [European Committee on Antimicrobial Susceptibility Testing (EUCAST), (2026)].

The same claim-based framework applies to planktonic and biofilm-associated populations, but the endpoints must be state-specific. Planktonic MIC changes cannot establish activity against a mature biofilm. Biofilm biomass, metabolic activity, viable-cell counts, MBIC or MBEC where operationally defined, and post-treatment regrowth address different outcomes (Ceri et al., 1999; Peeters et al., 2008). The review's report that cinnamaldehyde-colistin inhibited biofilm formation and dispersed preformed biofilms illustrates why stage and endpoint should be stated explicitly: prevention, biomass loss, dispersal, killing, and eradication are not interchangeable. Biomass or metabolic measurements should therefore be paired with viable-cell or regrowth assessment when killing or eradication is claimed. Clinical breakpoints established for planktonic susceptibility testing should not be applied to biofilm endpoints. Table 1 summarizes the terminology used for the different antibiotic-adjunct claims and the minimum supporting evidence for each.

**Table 1:** Terminology and minimum evidence for antibiotic-adjunct claims.

Claim	What the claim means	Minimum supporting evidence
Resistance reversal	A defined resistance phenotype is attenuated through a mechanism-related adjunct effect.	Baseline resistance, antibiotic activity with and without adjunct, and dependence on the relevant determinant; target engagement where feasible.
Antibiotic potentiation	The adjunct increases antibiotic activity without implying a specific mechanism or susceptibility category.	Dose-response data for both agents and comparison of the combination with each agent alone.
Tolerance modification	Killing is accelerated without necessarily changing the MIC.	Time-dependent survival or killing measurements under defined exposure conditions.
Persistence eradication	A non-heritable surviving subpopulation is reduced without a stable change in susceptibility.	Time-dependent survivor quantification, post-exposure regrowth assessment, and confirmation that regrown cells have not acquired a stable MIC shift.
Biofilm disruption or dispersal	Biofilm biomass, matrix, or architecture is reduced, or attached cells are released from a mature biofilm.	A structural or biomass endpoint; released-cell quantification or microscopy when dispersal is claimed; viable-cell and regrowth outcomes when killing or eradication is claimed; formation and mature-biofilm assays distinguished.
Direct antibacterial activity	The adjunct alone inhibits growth or kills bacteria.	Adjunct-alone dose-response at combination concentrations and analysis showing what the combination adds beyond the individual effects.

## Mechanistic validation and translation

Mechanistic support becomes progressively stronger when phenotypic potentiation is accompanied by dependence on the proposed resistance determinant in isogenic or otherwise well-controlled systems, biochemical or cellular target engagement, and orthogonal confirmation such as altered drug accumulation or enzyme activity. Loss of potentiation in a determinant-deficient strain is supportive but not conclusive because adjuncts may be pleiotropic. Intrinsic antibacterial activity also does not disqualify an adjunct; it requires the authors to distinguish combination antimicrobial therapy from a mechanism-dependent resistance-breaking effect.

This framework complements the translational challenges already identified by Nakazwe et al. Screening establishes interaction, mechanistic studies establish attribution, preclinical studies test relevant isolates and biofilm states, and translation requires simultaneous exposure of both agents at the infection site together with acceptable safety. Terminology should follow the evidence stage: candidate resistance-breaking adjunct at screening, antibiotic potentiator when enhancement is shown, and resistance-restoring combination only when the partner antibiotic, resistance phenotype, physiological state, susceptibility or killing outcome, and mechanism are specified.

## Discussion

Resistance breaker can remain a useful umbrella term. For individual studies, however, the claim should be attached to a defined combination and physiological state, and the supporting endpoint should match the proposed mechanism and level of translation. This wording preserves the breadth of the original review while separating synergy, tolerance modification, biofilm disruption, direct antibacterial activity, and demonstrated resistance reversal.
